# Morphological, microstructural and photocatalytic characterization of undoped and Ni, Co doped Fe_2_O_3_ particles synthesized by sonochemical method

**DOI:** 10.55730/1300-0527.3489

**Published:** 2022-08-02

**Authors:** Elif EMİL-KAYA, Burak EVREN, Zeynep ERDÖL, Duygu EKİNCİ, Mehmet İPEKOĞLU, Sibel ÖZENLER

**Affiliations:** 1 Department of Materials Science and Engineering, Faculty of Georesources and Materials Engineering, RWTH Aachen University, Aachen, Germany; 2 Department of Materials Science and Technology, Faculty of Science, Turkish-German University, İstanbul, Turkey; 3 Department of Metallurgical and Materials Engineering, Faculty of Chemical and Metallurgical Engineering, İstanbul Technical University, İstanbul, Turkey; 4 Department of Materials Science and Engineering, Faculty of Engineering, Gebze Technical University, Kocaeli, Turkey; 5 Department of Mechanical Engineering, Faculty of Engineering, Turkish-German University, İstanbul, Turkey

**Keywords:** Fe_2_O_3_, crystallite size, lattice strain, dislocation density, photocatalytic performance, Williamson-Hall analysis

## Abstract

In this study, an abundant and eco-friendly photocatalytic material, Fe_2_O_3_ particles were synthesized by sonochemical method. Morphological and microstructural investigations of synthesized undoped and Ni, Co-doped Fe_2_O_3_ particles were performed. The effect of particle morphology and microstructure on its photocatalytic performance was further investigated. Comparative studies for evaluating particle crystallite sizes were conducted by Williamson-Hall (W-H) method and modified Debye-Scherrer (MDS). Crystallite sizes and lattice strains of Fe_2_O_3_ induced by process parameters were calculated by W-H method based on uniform deformation model (UDM). The crystallite sizes of the synthesized powders were calculated in the range of 200 nm and 76 nm by Williamson-Hall analysis. In addition to structural investigation, dislocation density of the synthesized particles was calculated by Williamson-Smallman relation. Afterwards, photocatalytic performance of Fe_2_O_3_ particles was investigated in detail. The photodegradation of methylene blue solutions in the presence of light in 20 min with samples 3,4, and 5 in 20 min were 0.937, 0.896, and 0.855, respectively. Moreover, the photodegradation of methylene blue solution with sample 5 for 15, 30, and 45 min were 0.9, 0.828, and 0.757, respectively. A photocatalytic activity of 24.25% has been observed under optimum conditions for the time interval of 45 min.

## 1. Introduction

In recent years, pollution in wastewater, especially caused by dyes, has caused serious environmental problems. There are many organic pollutants and dyes dropped directly into water sources from the chemical, pharmaceutical and textile industries. Dyes are extensively used in various industries during material processing. It is reported that 10%–15% of consumed dyes are mixed with water sources. Dyes are mostly hazardous; therefore, it is very important for the environment to treat any sort of waste that contains these substances [1–3].

Recently, several water treatment methods have been developed. Degradation of organic substances by photocatalytic materials is a promising method for removing organic pollutants from wastewater. Semiconductor materials such as TiO_2_, Al_2_O_3_, Sb_2_O_3_, Sm_2_O_3_, Fe_2_O_3_, WO_3_, CeO_2_, V_2_O_5_ have been used as photocatalytic materials in degradation of organic dies [4–16]. Among these semiconductor materials, hematite (Fe_2_O_3_) is abundant in earth, inexpensive and environmentally friendly. Therefore, it is mostly used in gas sensors, solar cells, lithium-ion batteries, fuel cells. Hematite also functions as photocatalytic material since it is a semiconductor [17–21]. Fe_2_O_3_ particles have been synthesized in various morphologies through hydrothermal [22], coprecipitation [23], solvothermal [24], spray pyrolysis [25] and sol-gel methods [26]. Photodegradation of various organics under UV light or daylight by synthesized Fe_2_O_3_ particles has been researched in several studies. The effect of doping elements to on Fe_2_O_3_ bandgap and photocatalytic activity has also been investigated [27].

Sivakumar et al. synthesized Ni doped Fe_2_O_3_ nanoparticles by chemical precipitation method. Bandgap of semiconductor is calculated from UV-Vis analysis results and reduction in bandgap with Ni doping was observed. Changes in the magnetic hysteresis curves with doping of nickel at different rates were investigated [28]. Transition element (Cu, Ni, Co) doped Fe_2_O_3_ nanoparticles for photocatalytic applications were synthesized by Satheesh et al. Magnetic and photocatalytic properties of synthesized powders were investigated. It was reported that acid red 27 (AR27) degradation rate of Cu doped Fe_2_O_3_ particles was the highest. As solution pH is among important factors that affect photodegradation, photodegradation activity of particles synthesized at pH levels between 3 and 9 was investigated. Highest photodegradation degree of 98.05% was reported at pH 6. Effect of catalyst and organic die concentrations was also investigated [29]. Sn doped Fe_2_O_3_ particles were synthesized by Mansour et al. through coprecipitation method. Sn was doped at molar concentrations of 0.01%, 0.03%, and 0.06%. Reduction in crystallite size and bandgap was reported. Highest Rhodamine B (RhB) degradation rate was reported for 0.06% Sn doped Fe_2_O_3_ particles [30].

In many studies in the literature, the effect of Fe_2_O_3_ morphology on photocatalytic activity was investigated. In the study of Yan et al., Rhodamine (RhB) degradation of Fe_2_O_3_ particles synthesized in the form of rings was investigated under UV light in the 400–800 wavelength range [31]. In another study, photocatalytic activity of nanoplate shaped hematite particles synthesized through microwave assisted solvothermal method was investigated against salicylic acid by Sun et al. [32]. By using different precipitating agents (NaCl, NaBr), Wang et al. synthesized Fe_2_O_3_ particles and investigated the difference in photocatalytic activity. Particles synthesized by NaCl showed higher surface area, therefore had a higher light absorbance and photocatalytic activity was increased [33]. Photocatalytic activity of hematite produced by sol-gel method at different calcination temperatures was investigated by Boumaza et al. Calcination temperature affected the surface area and therefore altered the photochemical activity [34].

In recent years, ultrasonic waves have been preferred in many studies to synthesize different materials. When liquids are exposed to strong sound waves, high- and low-pressure waves are formed, which causes small air bubbles to form, and when the bubbles reach a certain size, they split immediately. Current and turbulence emerged this way provides on one hand a strong agitation, on the other hand a homogeneous mixture is obtained. Moreover, turbulence splits the agglomerated particles and therefore is very effective in shrinking soft and hard particles [35]. It was reported that usage of ultrasonic waves in the synthesis process instead of conventional mixing and heating methods shortened the synthesis duration.

In this study, undoped, Ni and Co-doped Fe_2_O_3_ particles were synthesized through various process parameters via sonochemical method. The effect of process parameters including calcination time, the amount of ethylene glycol, the addition of PVP, and Ni, Co doping on photocatalytic activity of Fe_2_O_3_ was revealed. The particle morphologies of the synthesized hematite particles were manipulated by process parameters. The synthesized particles phase analysis was conducted by XRD. On the basis of XRD peaks, crystallite sizes were calculated by mathematical models including W-H and MDS methods. Moreover, dislocation densities were calculated by Williamson-Smallman model. In order to investigate the short-term photocatalytic activity of the synthesized particles, photocatalytic tests were conducted by methylene blue (MB) solution under a halogen light for different durations. The photodegradation of organic dyes was characterized by a UV-Vis spectrophotometer.

## 2. Materials and methods

Firstly, ethylene glycol was prepared. Afterwards, 1% wt polyvinylpyrrolidone (PVP, mn: 10000) was dissolved in ethylene glycol in an ultrasonic bath. The base solution was prepared using a 200 mL ethylene glycol – PVP mixture and 100 mL deionized water. Iron nitrate, nickel nitrate and cobalt nitrate (Fe(NO_3_)_3_.6H_2_O, Ni(NO_3_)_3_.6H_2_O and Co(NO_3_)_3_.6H_2_O Carlo Erba) solutions were prepared. Then, iron nitrate (0.1mol/L) solution was prepared by mixing of nickel nitrate (vol. 1.5%) and cobalt nitrate (vol. 1.5%) for producing of Ni, Co doped Fe_2_O_3_. Afterwards, gelation was initiated by adding ammonia (NH_4_OH, Merck) to iron nitrate solution. After the beginning of gelation, solution was mixed with an ultrasonic homogenizer for 6 min. After 48 h, precipitates were filtrated and dried in the oven for 24 h. Different samples were calcined at 700 °C and 900 °C for 3 h. After calcination, Fe_2_O_3_ formation was observed. Experimental parameters are given in Table 1. Crystal structures of synthesized particles were analyzed by X-ray Diffractometer (Bruker AXS/Discovery D8) with monochromatic CuKα tube in the range of 10–90° within 0.02° steps and phase analyzes were conducted by X’Pert HighScore program. The effect of process parameters on crystallite sizes was conducted by Williamson-Hall analysis based on uniform deformation model. In order to compare the results obtained by Williamson-Hall analysis, the modified Debye-Scherrer method was also used to calculate crystallite sizes. Dislocation density was calculated by Williamson-Smallman analysis from the calculated crystallite sizes. In order to observe the effect of process parameters on powder morphologies, synthesized particles were analyzed by field emission gun scanning electron microscopy (FEG-SEM, Philips XL30). Methylene blue solutions (10 mg/L) were prepared to investigate the photocatalytic performances of synthesized particles. These solutions were mixed for 30 min in a dark box to reach absorption-desorption balance. Afterwards, hematite particles were added to methylene blue solutions with a ratio of 5 g/L. Fifty milliliters of suspension was mixed under a 100-W halogen lamp with a wavelength range of 350–1050 nm at a 15-cm distance. Samples were repeatedly taken from suspension during mixing to analyze the effect of exposure time. The catalyst material was separated from the suspension by centrifuge (Hettich, Rotina 420R). Spectral analysis was conducted by UV-Vis spectrometer (Analytikjena, Specord 200 Plus).

## 3. Results and discussion

### 3.1. Microstructural investigation of undoped and Ni, Co doped Fe_2_O_3_ particles

**Figure 1:**
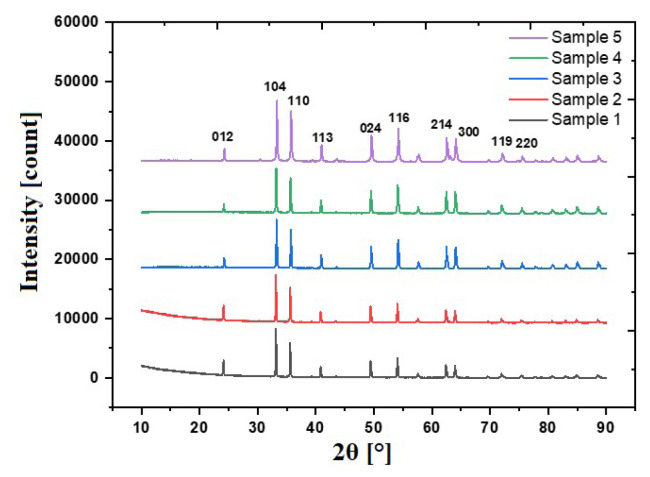
XRD patterns of samples 1–5.

Phase analyzes of undoped and Ni, Co-doped Fe_2_O_3_ particles synthesized under different conditions were conducted and XRD patterns are given in Figure 1. Reference patterns of synthesized particles were determined as 01-084-0307 by X’Pert HighScore Plus software. The crystal structure of synthesized particles belongs to the rhombohedral crystal system and the space group is R-3c. Despite the changes in the production parameters, it was observed that the crystal system did not change. The determined calcination temperatures were suitable for Fe_2_O_3_ crystallization. Effect of process parameters on crystal sizes was conducted by Williamson-Hall analysis based on uniform deformation model. Initially, 7 peaks with high diffraction intensities were selected and peak widths were determined by X’Pert High Score Plus software. Strain-induced peak broadenings are considered as crystal defects and shown as ɛ = βs/tanθ. The equations below are derived from Scherrer and ɛ = βs/tanθ equations. Peak broadening was calculated by subtracting instrumental peak broadening from measured peak broadening as shown in Equation 1.


(1) $$\beta_{hkl}={\left[\beta_{measured}^{2}-\beta_{instrumental}^{2}\right]}^{\frac{1}{2}}$$

Peak broadening consists of crystallite size and crystallite strain as shown in Equation 2.


(2) $$\beta_{hkl}=\beta_{crystallite}+\beta_{strain}$$

Crystal distortion is formulated with Equation 3.


(3) $$ɛ\approx \beta_{\text{s}}/\text{tan}\theta$$

Equations 5 and 6 are derived by using Debye-Scherrer equation and equations above.


(4) $$\beta_{hkl}=\left(\frac{k\lambda }{Dcos\theta }\right)+(4ɛ\;\text{tan }\theta )$$

(5) $$\beta_{hkl}cos\theta =\left(\frac{k\lambda }{D}\right)+(4ɛ\;\text{sin }\theta )$$

β*_hkl_* denotes peak broadening, θ denotes diffraction angle, whereas k denotes shape factor (in this case: 0.84), λ is the wavelength of *CuKα* radiation (λ= 0,154184 nm), *D* denotes average crystallite size and ɛ is average lattice strain.

β_hkl_cosθ - 4ɛsinθ graph is plotted for calculating crystallite size and strain. Values were subjected to linear fitting. The slope of the line equals strain. The point where the line intercepts the y-axis equals crystallite size.

**Figure 2:**
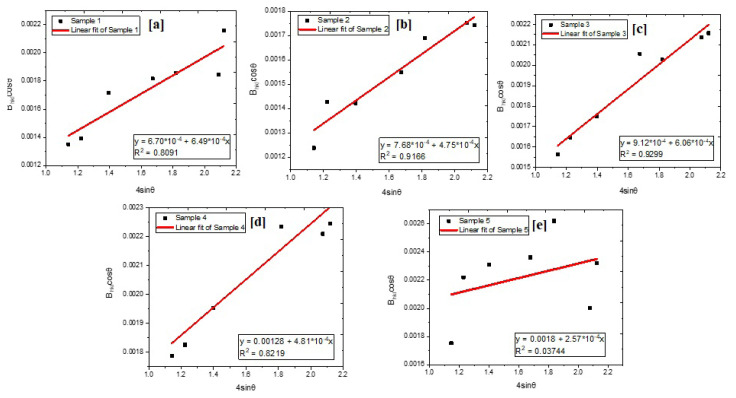
Williamson-Hall analyses of (a) sample 1, (b) sample 2, (c) sample 3, (d) sample 4, and (e) sample 5.

Graphs plotted by Williamson-Hall analysis are given in Figure 2.

**Figure 3:**
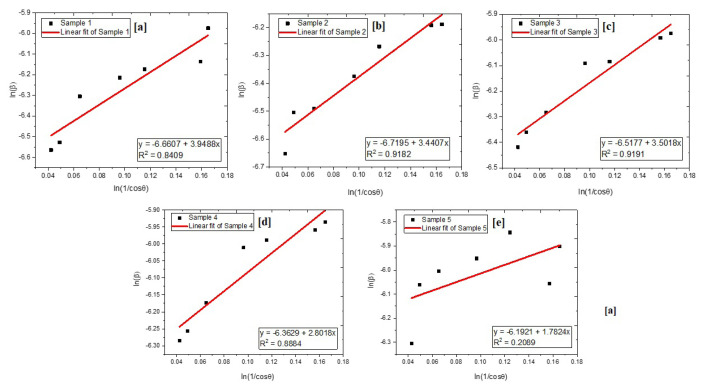
Modified Debye-Scherrer analyses of (a) sample 1, (b) sample 2, (c) sample 3, (d) sample 4, and (e) sample 5.

Williamson-Hall analysis based on uniform deformation model revealed that crystallite sizes were 204, 178, 150, 107, and 76 nm, indicating that nanostructured Fe_2_O_3_ particles were obtained. After Ni and Co doping, a reduction in crystallite size was observed. Moreover, PVP usage reduced agglomeration during calcination and crystallite sizes were decreased with increased PVP amount. The decrement in crystallite size was observed when calcination temperature was lowered from 900 °C to 700 °C. Similar observations were reported in previous studies [36,37]. Modified Debye-Scherrer analysis was conducted to compare the results of Williamson-Hall analysis. Graphs of modified Debye-Scherrer analysis are given in Figure 3.

According to the modified Debye-Scherrer analysis, crystallite sizes of samples were calculated as 113, 107, 92, 79, and 67 nm. Retrieved results were relatively smaller and in accordance with Williamson-Hall’s analysis. As expected, crystallite size values evaluated by the MDS method differ from the ones calculated by the W-H method owing to the negligence of the lattice strain. Due to the presence of tensile stress in the lattice, MDS method calculates smaller crystallite sizes than the W-H method. In case of compressive stresses, MDS method calculates higher crystallite sizes than the W-H method. Similar observations were reported in previous studies [38–42].

Dislocation densities were calculated by Williamson-Smallman analysis from crystallite sizes obtained by Williamson-Hall analysis. Williamson-Smallman equation is given in Equation 6.


(6) $$\delta =\left(\frac{1}{D^{2}}\right)$$

Calculated dislocation densities and crystallite sizes are given in Table 2.

An increment in dislocation density with reduced crystallite size was observed.

### 3.2. Investigation of powder morphologies of undoped, Ni and Co-doped Fe_2_O_3_ particles

**Figure 4:**
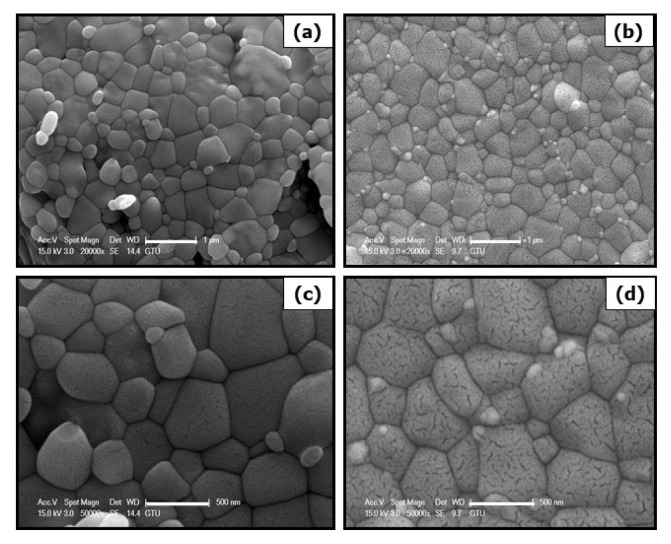
SEM images with secondary electrons of synthesized samples (a), (c) undoped Fe_2_O_3_ (sample 1) and (b), (d) Ni, Co doped Fe_2_O_3_ (sample 2).

In order to investigate the effects of process parameters on powder morphology, scanning electron microscopy imaging was conducted. Figure 4 represents SEM images with secondary electrons images of undoped, Ni and Co-doped Fe_2_O_3_ particle.

**Figure 5:**
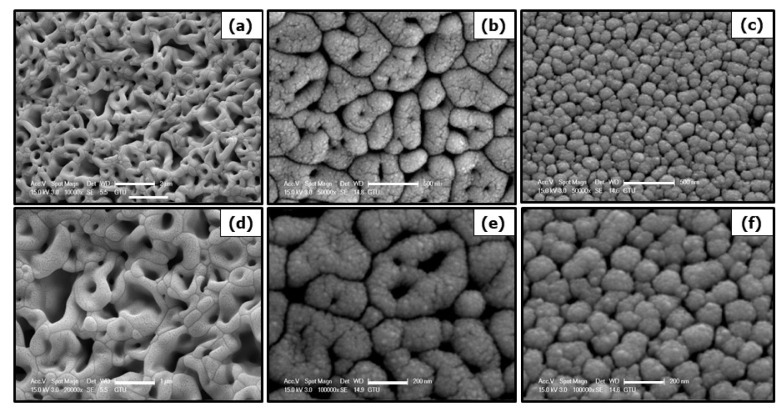
SEM images with secondary electrons of synthesized samples (a), (d) sample 3 (b), (e) sample 4 and (c), (f) sample 5.

From scanning electron microscopy images, it was observed that synthesized Fe_2_O_3_ particles were agglomerated during the calcination step. In Ni and Co-doped samples, linear voids on grains were caused by doping elements. PVP agent was added and calcination temperature was decreased for reducing agglomeration. In order to compare the effect of PVP addition and calcination temperature, samples 3, 4, and 5 were imaged by scanning electron microscopy at different magnifications. Figure 5 illustrates SEM images with secondary electrons images of samples 3, 4, and 5.

Ethylene glycol and PVP were used to prevent to agglomeration of particles. In particular, PVP was used to encapsulate particles. Samples synthesized by the sonochemical route and contained PVP were not agglomerated and had a porous structure. Agglomeration behavior was not observed when the calcination temperature was lowered. Moreover, at lower calcination temperatures, samples obtained a spherical morphology.

### 3.3. Investigation of photocatalytic performance of undoped, Ni and Co doped Fe_2_O_3_ particles

In order to investigate the effect of powder morphology and microstructure on photocatalytic performance, photocatalysis experiments were conducted comparatively at different durations.

**Figure 6:**
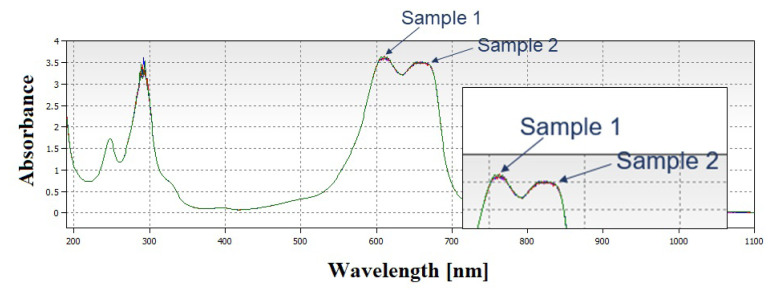
UV-Vis spectrum of MB and photocatalysis experiment results of samples 1 and 2.

Initially, photocatalysis experiments of agglomerated particles were conducted and their results are given in Figure 6.

**Figure 7:**
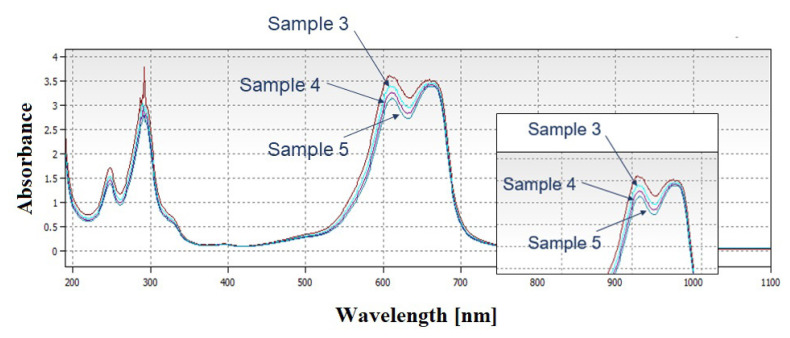
UV-Vis spectrum of MB and photocatalysis experiment results of samples 3, 4, and 5.

According to UV-Vis spectrums of samples 1 and 2, particles did not exhibit photocatalytic performance. These particles were proven to be agglomerated from SEM imaging and agglomeration was considered to affect photocatalytic performance dramatically. In order to investigate the photocatalyst performances of not agglomerated particles, comparative UV-Vis spectrums of samples 3, 4, and 5 were plotted and results are given in Figure 7.

**Figure 8:**
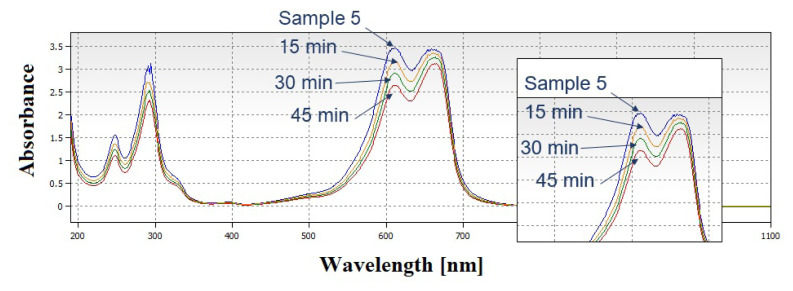
UV-Vis spectrum of MB and photocatalysis experiment results of sample 5 exposed to light for 15, 30, and 45 min.

Samples 3, 4, and 5 were subjected to photocatalysis experiments for 20 min. The best performance was obtained from sample 5, which was not agglomerated during the calcination step. Investigating the SEM images and XRD patterns, sample 5 had a lower particle size than other samples. According to Williamson-Hall analysis, sample 5 had the lowest crystallite size. As sample 5 showed the highest photocatalytic activity, in order to investigate the effect of light exposure duration, it was exposed to halogen light for 45 min and changes in the absorbance with time is measured by UV-Vis spectrometer. UV-Vis spectrum is given in Figure 8.

An increment in methylene blue degradation was observed with a prolonged photocatalytic activity experiment. The effect of particle morphology and microstructural properties on photocatalytic performance was investigated and although the crystallite sizes were able to be decreased by doping elements, agglomerated particles did not show photocatalytic activity. Particles with porous shapes that were not agglomerated exhibited highest photocatalytic activity.

**Figure 9:**
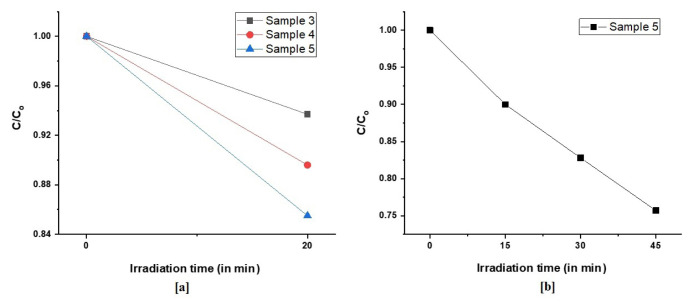
(a) Photodegradation of MB in the presence of light by samples 3, 4, and 5 for 20 min. (b) Photodegradation of MB in the presence of light by sample 5 for 15, 30, and 45 min.

The photodegradation of MB in the presence of light by samples 3, 4, and 5 for 20 min was calculated. Figure 9a, represents the photodegradation of MB in the presence of light by samples 3, 4, and 5 for 20 min. Figure 9b illustrates the photodegradation rate for time interval of 45 min.

**Figure 10:**
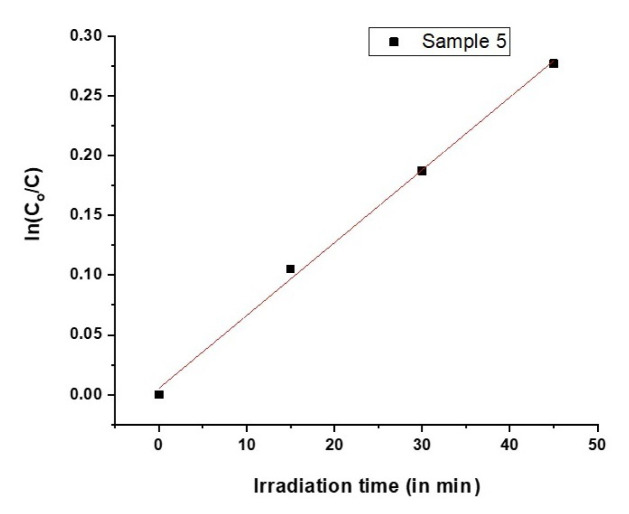
Linearly fitted curve of ln(C_0_/C) as a function of time for sample 5.

From the photodegradation results of sample 3,4, and 5, it is clear that sample 5 shows higher photocatalytic rate than samples 3 and 4. Therefore, sample 5 was chosen for the prolonged photodegradation experiment. Moreover, the photodegradation of methylene blue solution with sample 5 for 15, 30 and 45 min were 0.9, 0.828, and 0.757, respectively. Figure 10 shows linearly fitted curve of ln(C_0_/C) as a function of irradiation time for sample 5.

A linear behavior of Figure 10 shows the pseudo first order degradation kinetics. The slope of linear fit of this graph is 0.006, this value indicates the reaction constant.

The efficiency of the photocatalytic activity is calculated from the formula:


$$\text{Rate of degradation}=\frac{{\text{A}}_{\left(\text{i}\right)}-{\text{A}}_{\left(\text{t}\right)}}{{\text{A}}_{\left(\text{i}\right)}}\times 100$$

Table 3 illustrates the efficiency of the degradation of MB by samples 3, 4, and 5 in 20 min.

The efficiency of photodegradation of MB by samples 3, 4, and 5 were 6.20%, 10.30%, 14.50%, respectively, in 20 min. Table 4 represents the efficiency of the degradation of MB by sample 5 for the time interval of 45 min.

Sample 5 exhibited the photocatalytic activity with 10%, 17.15%, and 24.25% for the durations of 15, 30, and 45 min, respectively. In particular, sample 5 exhibited the better MB degradation property with 24.25% efficiency in 45 min.

Keerthana et al. investigated the photocatalytic efficiencies of Fe_2_O_3_, 2% Co-doped Fe_2_O_3_, and 4% Co-doped Fe_2_O_3_. The 4% Co- Fe_2_O_3_ sample illustrated the better dye degradation with 92% efficiency in 120 min. Moreover, the photocatalytic efficiency of Fe_2_O_3_, 2% Co-doped Fe_2_O_3_, and 4% Co-doped Fe_2_O_3_ were approximately 10%, 16.6%, and 30% in 40 min, respectively. Increasing the amount of cobalt favored the increase of the photocatalytic activity of the samples [43]. In another study, Ilkme et al. investigated the effect of transition metals such as Co, Ni, and Cu on the photocatalytic activity of Fe_2_O_3_-V_2_O_5_ binary oxide. The addition of transition metal improved the photocatalytic activity of the binary oxide and the addition of Cu exhibited the highest percentage of 2,4-dichlorophenol degradation (100%) in 30 min. Particularly, compared to the photocatalytic activity of pure Fe_2_O_3_, the improved photocatalytic activity of Cu doped Fe_2_O_3_-V_2_O_5_ could be ascribed to the addition of Cu and V_2_O_5_ [44]. Gu et al. studied the effect of the addition of PVP on the photocatalytic activity of TiO_2_, the presence of PVP in the sol-gel process has been proved to be efficient in the photodegradation of methylene red under UV light [45]. Similarly, Phuruangrat et al. evaluated the effect of weight contents of PVP in the solutions on the synthesized photocatalytic BiOCl powders. The synthesized BiOCl nanoplate powders with PVP addition exhibit photodegradation of Rhodamine B (RhB) under visible light irradiation compared to BiOCl nanoplates without PVP addition. The weight content of PVP was ascribed to play a significant role in the morphology and size of BiOCl powders. BiOCl nanoplate powders with PVP addition exhibited the best photocatalytic efficiency for RhB photodegradation of 97.61% in 240 min [46]. Similarly, in our work, the addition of PVP plays a significant role in the photodegradation of MB and Fe_2_O_3_ powders with PVP addition showed the best degradation efficiency of MB.

## 4. Conclusion

In this study, undoped Fe_2_O_3_ and Ni and Co-doped Fe_2_O_3_ particles were synthesized from Fe(NO_3_)_3_ solution via sonochemical method. SEM image of the synthesized particles revealed that samples 1 and 2 were agglomerated during calcination. Samples 3, 4, and 5 had a porous structure due to PVP addition since PVP encapsulated the synthesized particles. The change in the calcination temperature from 900°C to 700°C prevented the agglomeration. The crystal structure of particles was identified as rhombohedral by XRD analysis. In order to investigate the effect of process parameters on microstructural properties, Williamson-Hall, modified Debye-Scherrer analyzes were conducted based on the XRD peak broadenings. The crystallite size of agglomerated sample 1 was calculated at 204 nm, in Co and Ni-doped samples, crystallite size is reduced as cobalt and nickel atomic radii were smaller than iron. PVP addition during synthesis prevented agglomeration and reduced the crystallite sizes. Moreover, lowering the calcination temperature prevented the formation of tensile stresses in lattice and crystallite sizes were reduced. The crystallite size of sample 5 was calculated at 76 nm. The crystallite sizes were also calculated by modified Debye-Scherrer analysis and as this calculation did not involve lattice strains, results differed from Williamson-Hall analysis, however, the results were parallel for both methods. The dislocation densities were calculated by Williamson-Smallman analysis. An increment in dislocation density was observed with reduced crystallite size. The agglomerated particles with reduced crystallite size by doping elements still did not show photocatalytic activity. Particles with a porous shape exhibited the highest photocatalytic activity. The photodegradation of MB solutions in the presence of light in 20 min with samples 3, 4, and 5 in 20 min were 0.937, 0.896, and 0.855, respectively. Moreover, the photodegradation of MB solution with sample 5 for 15, 30, and 45 min were 0.9, 0.828, and 0.757, respectively. A photocatalytic activity of 24.25% has been observed under optimum conditions for the time interval of 45 min.

## Figures and Tables

**Table 1:** Experimental parameters.

Sample	Co:Ni dop	PVP (gr)	Ethylene glycol (mL)	Cal. temp (°C)	Homogenization time (min)
Sample 1	−	−	16	900	6
Sample 2	+	−	16	900	6
Sample 3	−	1.5	100	900	6
Sample 4	−	1.5	100	700	6
Sample 5	−	3	100	700	6

**Table 2:** Results of W-H and W-S analyzes.

Sample	W-H analysis (nm)	W-S analysis (δ)
Sample 1	204	2.40 × 10^−5^
Sample 2	178	3.15 × 10^−5^
Sample 3	150	4.44 × 10^−5^
Sample 4	107	8.73 × 10^−5^
Sample 5	76	1.73 × 10^−4^

**Table 3:** The efficiency of the photodegradation of samples 3, 4, and 5 in 20 min.

Sample	Photocatalytic efficiency in 20 min (%)
Sample 3	6.20
Sample 4	10.30
Sample 5	14.50

**Table 4:** The efficiency of the degradation of MB by sample 5 for the time interval 45 min.

	Photocatalytic efficiency (%)		
	15 min	30 min	45 min
**Sample 5**	10	17.15	24.25
